# Variation in bone response to the placement of percutaneous osseointegrated endoprostheses: A 24-month follow-up in sheep

**DOI:** 10.1371/journal.pone.0221850

**Published:** 2019-10-25

**Authors:** Sujee Jeyapalina, James Peter Beck, Alex Drew, Roy D. Bloebaum, Kent N. Bachus

**Affiliations:** 1 Research, Department of Veterans Affairs Medical Center, Salt Lake City, Utah, United States of America; 2 Division of Plastic Surgery, Department of Surgery, University of Utah School of Medicine, The University of Utah, Salt Lake City, Utah, United States of America; 3 Department of Orthopaedics, University of Utah Orthopaedic Center, University of Utah School of Medicine, Salt Lake City, Utah, United States of America; 4 Department of Bioengineering, University of Utah College of Engineering, The University of Utah, Salt Lake City, Utah, United States of America; 5 Orthopaedic Research Laboratories, Department of Orthopaedics, University of Utah School of Medicine, Salt Lake City, Utah, United States of America; 6 Bone and Joint Research Laboratory, Department of Veterans Affairs Medical Center, Salt Lake City, Utah, United States of America; Universidad de Zaragoza, SPAIN

## Abstract

Percutaneous osseointegrated **(OI)** devices for amputees are metallic endoprostheses, that are surgically implanted into the residual stump bone and protrude through the skin, allowing attachment of an exoprosthetic limb. In contrast to standard socket suspension systems, these percutaneous OI devices provide superior attachment platforms for artificial limbs. However, bone adaptation, which includes atrophy and/or hypertrophy along the extent of the host bone-endoprosthetic interface, is seen clinically and depends upon where along the bone the device ultimately transfers loading forces to the skeletal system. The goal of this study was to determine if a percutaneous OI device, designed with a porous coated distal region and an end-loading collar, could promote and maintain stable bone attachment. A total of eight, 18 to 24-month old, mixed-breed sheep were surgically implanted with a percutaneous OI device. For 24-months, the animals were allowed to bear weight as tolerated and were monitored for signs of bone remodelling. At necropsy, the endoprosthesis and the surrounding tissues were harvested, radiographically imaged, and histomorphometrically analyzed to determine the periprosthetic bone adaptation in five animals. Bone growth into the porous coating was achieved in all five animals. Serial radiographic data showed stress-shielding related bone adaptation occurs based on the placement of the endoprosthetic stem. When collar placement and achieved end-bearing against the transected bone, distal bone conservation/hypertrophy was observed. The results supported the use of a distally loading and distally porous coated percutaneous OI device to achieve distal host bone maintenance.

## Introduction

Percutaneous osseointegrated **(OI)** devices have been utilized as an alternative to socket suspension of prosthetic limbs for patients with amputations [1–5]. Over the past two decades, many investigators worldwide have bypassed translational animal research studies, commonly testing and changing device designs as part of an empirical surgical approach to skeletal limb attachment [1–6]. While this method has the benefit of expedited clinical results, it has the concomitant shortcomings of “trial and error” techniques and the potential for patient morbidity. Although this has given rise to multiple device designs, surgical techniques, clinical protocols, and rehabilitation strategies, little is known about the actual bone-endoprosthetic attachment sites, the amount of osseointegration that might be occurring, or how the bone adapts to these devices.

In the current clinical applications, intramedullary osseointegration is accomplished by either screwing a pure titanium “fixture” into the tapped medullary canal (OPRA, Swedish design [2, 3, 7]) or driving a press fitted stem into the prepared femoral shaft (Osseointegrated Leg Prosthesis or OILP, German design [4, 8, 9]). In the case of the OPRA device, osseointegration occurs because of intimate and tight surface contact of the pure titanium screw threads of the “fixture” against the adjacent cortical bone. This device is similar to those used for dental devices. The OPRA device is comprised of an 8 cm long endoprosthesis and is implanted using a two-stage surgical procedure. During the first surgery, the endoprosthesis is proximally countersunk 2 cm deep to transected end of the femur. This empty 2 cm deep cylinder at the end of the bone is then filled with cancellous bone graft material. Since this 2 cm portion is not loaded, with increasing time *in situ*, this unloaded bone appears to resorb, and within 2-years post implantation, published radiographs show severe resorption of this 2 cm region [10]. The OILP design is also not loaded at the resected end. Here, the distal end of the endoprosthesis is tapered and uncoated, and the osseointegration surface is long, extending almost the entirety of the endoprosthesis [4, 8, 11]. This allows immediate secure press-fit fixation and a larger surface area for osseointegration. Sometimes, imaging of these patients shows distal regional resorption. Also, in the event of periprosthetic infection, and the need for device removal, this length of osseointegrated bone risks a substantial loss of host bone stock.

In order to circumvent these outcomes, an end-loading endoprosthesis was designed. Research conducted jointly at the VA Salt Lake City Health Care System and at the University of Utah Orthopaedic Center has shown that a porous commercially pure titanium coating provided a stable integration of bone with a titanium-alloy endoprosthesis [12–16]. The design consisted of a line-to-line endoprosthesis that fit the surgically prepared medullary canal of the transmetaphyseal-amputated, fused 3–4 metacarpal of the sheep forelimb [12, 16]. Sheep were chosen for the investigation [12, 16], because they provided a translational large animal model with both body weight and bone remodeling rate comparable to those of an adult human [17]. The forelimb was chosen as the site for implantation because the standing orientation of the forelimb is more vertical than that of the hind-limb [18]. It should be noted that the skin-device interface analysis of this sheep study was previously published [19], and this present study only reports the bone ingrowth and remodeling outcomes.

The translational large animal model provided the opportunity to measure the amount of bone ingrowth into the porous coating of the endoprosthesis at 0-, 3-, 6-, 9-, and 12-months [15]. The rate of bone ingrowth over time was shown to be statistically highest at the beginning of the study (0- to 3-months) then decreased over time and began to plateau between 9- and 12-months [15]. In term of translational animal studies, this 12-month study was meant to be a relatively longer-term study. As the bone remodeling was a slow process, there was no statistical evidence to suggest that by 12-months, the rate of bone ingrowth had plateaued. Furthermore, in some animals, in which distal loading was prevented by proximal binding of the device in the diaphyseal canal, periprosthetic bone showed higher degrees of distal bone resorption [15, 16]. This occurred because a limited number of device sizes sometimes prevented ideal matching of the endoprosthesis to the configuration of the medullary canal. Thus, the present study was conducted, using the established translational large animal model [12, 14, 20], to determine the ability of a press-fit, distally porous coated, percutaneous OI endoprosthesis to achieve distal skeletal attachment and distal bone maintenance over a 24-month period. Using similar experimental conditions to those previously reported [15], the tested hypothesis was that the 1) bone ingrowth and 2) modeling and remodeling of percutaneous OI devices *in-situ* for 24-months would not be significantly different from those devices in-situ for 12-months.

## Materials and methods

### Endoprosthetic design and fabrication

**Fig 1:**
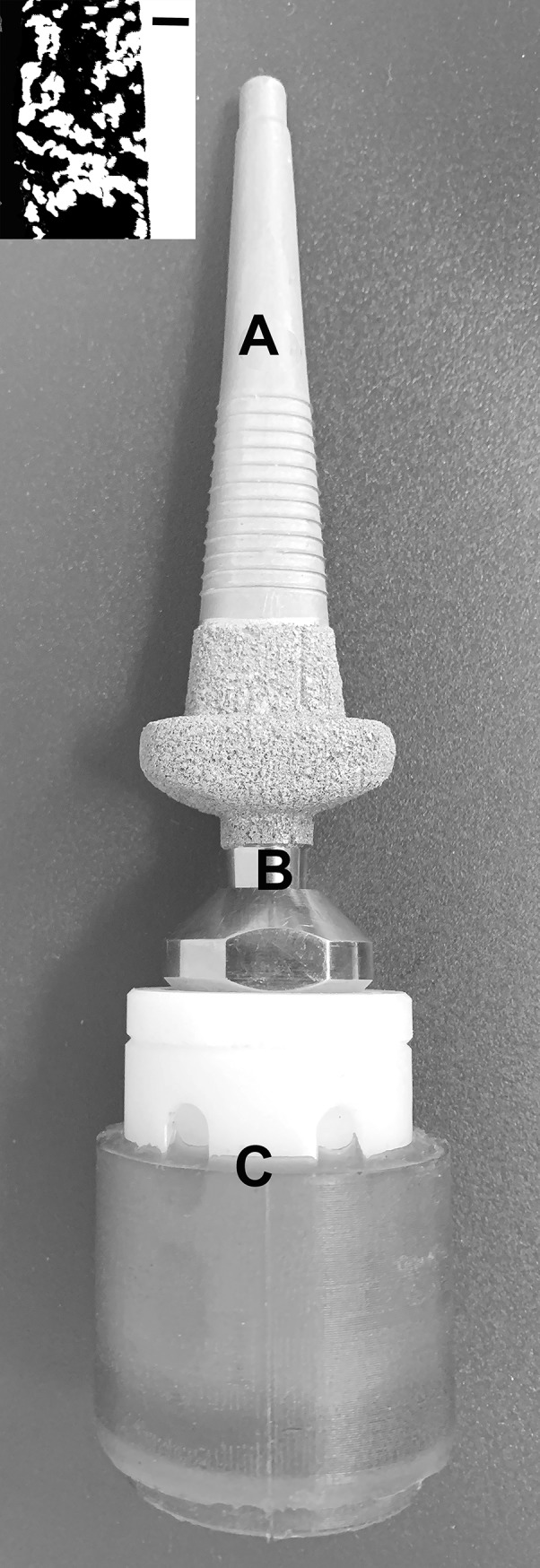
A photograph of the sheep percutaneous OI device showing endoprosthesis (A), percutaneous post (B) and exoprosthetic hoof (C). Inset is the cross-sectional electron backscattering image showing the thickness of the porous commercially pure titanium coating (~1 mm). White–metal. Black–pores. Scale bar—300 μm.

The endoprosthetic design and instrumentation used for this study has been previously published [19]. Briefly, endoprosthesis were fabricated from commercial grade titanium alloy, and the intramedullary surface was textured by grit blasting to facilitate bone attachment **(Fig 1)**. The subdermal barrier portion and 10 mm of the distal stem were coated with a 500–750 μm thick commercially pure titanium porous coating (P2, DJO Global, Austin, TX) with a reported porosity of 52 ± 12% [16]. The endoprosthesis incorporated a bead-blasted proximal stem, a 10mm long porous coated distal region for providing skeletal attachment, and a porous coated collar for loading the end of the resected bone [15].

### Study design and inclusion criterion

Eight sheep (n = 8) underwent amputation and percutaneous OI implantation surgery. Only animals that survived the entirety of the study were included in this bone ingrowth and adaptation study. Also, in order to limit the variabilities associated with the surgical procedure, a single surgeon performed all surgeries and followed an IACUC protocol. Three institutional approvals were obtained: 1) Innovative Medical Device Sourcing (IMDS) discovery Research, 2) University of Utah and 3) Department of Defense Animal Care and Use Review Office.

### Surgery

The surgical procedure mirrored our previous publications [12, 19, 21]. Briefly, after obtaining Institutional approvals (University of Utah, Department of Defense Animal Care and Use Review Office, and Innovative Medical Device Sourcing Discovery Research), eight, skeletally mature, 18- to 24-month old sheep of mixed breeds (Rambouillet, Targhee, Crossbred) were used. Pre-operative and intra-operative analgesic therapy was administrated to control pain, which included a 50 μg/hr Fentanyl patch 12-hours prior to surgery, and 3 mg/kg Ketoprofen and 1ml Buprenorphine immediately prior to surgery. An anteriorly based skin flap was established to remove the “dew-claws” of the foot. In each animal, the right metacarpal 3–4 bone was amputated at the epiphyseal/metaphyseal junction and the hoof flexor and extensor tendons were tenodesed to the periosteum with FiberWire sutures (Arthrex Vet Systems, Naples, FL). The intramedullary canal was then surgically prepared for press-fitting of the endoprosthesis using various sized broaches. An optimally sized endoprosthesis was then selected to achieve a tight fit in each animal [12, 19, 21]. After broaching the medullary canal and using frequent saline irrigations to prevent heat necrosis of the bone, the endprosthetic portion of the device was carefully driven into the medullary canal, and the exoprosthetic male Morse taper was then allowed to protrude though a sagittally oriented skin opening in the anterior based flap. Following surgical wound closure, a moderately compressive wrap (Vetrap®, Animal Care Products, 3M, St. Paul, MN) was used to protect the surgical site for 2 weeks.

### Post-operative care

Postoperative care also mirrored our previous publications [12, 19, 21]. Post-surgery, the animals were allowed to immediately bear weight as tolerated and kept indoors for 2 weeks. They were then housed in a covered barn with hay-covered concrete flooring. Post-operatively, analgesic treatment included an intramuscular injection of Ketoprofen (5.0 mg/kg) each day for seven days and a transdermal Fentanyl patch delivering a dose of 50 μg/hr for 9 days. Additional analgesics were administered at the discretion of the attending veterinarian.

Periprosthetic sites were cleaned with antimicrobial spray and water and forelimb functions were evaluated frequently searching for possible infection and lameness. This was done by either a trained animal care technician, or by a veterinary surgeon. Using a portable radiographic unit (PXP-40HF; United Radiology Systems Inc., Deerfield, IL), the bone response to the presence of the percutaneous OI device was monitored at 6-month intervals. At each time point, anteroposterior (AP) and mediolateral (ML) radiographs were taken.

### Necropsy

Two years following amputation and implantation of the percutaneous OI devices, or sooner if infected, the animals were euthanized using the approved procedure by the American Veterinary Medical Association. At necropsy, the implanted limbs were disarticulated through the proximal carpal joint and transported to the Department of Veterans Affairs Salt Lake City Healthcare System, Salt Lake City, UT for further processing.

### Analyses

All implanted specimens were subjected to radiographic imaging (Torrex 120D; Scanray, Hawthorne, CA) at 70 kV for 20 seconds, and selected specimens (n = 4) were also subjected to microcomputed tomography (μ-CT) scans. A standalone μ-CT system (Quantum FX, PerkinElmer, Hopkinton, MA) was used at 90 kVp, 160 mA tube current, and a 2-minute acquisition time for improved signal-to-noise ratio in the images. Three separate, but overlapping, scans were needed to image the entire metacarpal bone. At the detector, a ring reduction filter was used to reduce the metal artifacts caused by the presence of the titanium endoprostheses. Overlapping μ-CT image stacks were aligned and merged using the medical image processing software package Amira 6.0 (FEI, Hillsboro, OR). The endoprosthetic stem of the endoprosthesis was then segmented and used to align the image stack along the endoprosthesis axis. Once aligned, coronal and transverse cross-sectional images were extracted to evaluate bone-endoprosthesis contact and inter-digitation with the metaphyseal and endosteal cortical bone within the porous surface.

Following completion of the μ-CT, the devices and surrounding tissue were fixed in 10% formalin phosphate buffer, dehydrated in ascending grades of ethanol, and infiltrated and embedded in PMMA using an established procedure [22]. Once polymerized, 2-3mm thick sections were cut from each polymerized block using a custom-designed water-cooled, high-speed cut-off saw. The sequential bone-endoprosthesis sections were radiographed, and then were ground and polished to an optical finish.

The ground and polished sections of the bone-endoprosthesis were then imaged at X100 magnification using a scanning electron microscope (JSM 6100; JEOL Inc., Peabody, MA) equipped with a backscattered electron (BSE) detector (JEOL-64090BEIW; JEOL Inc., Peabody, MA). Commercially available software (IQ Materials System, Version 2; MediaCybernetics, Bethesda, MD) was used for evaluation. Grayscale threshold values of 0 to 5 represented the pores (black), 6 to 245 represented the bone tissue (gray), and 246 to 255 represented device material (white). The percentage of bone within the porous-coated region was represented as the area occupied by bone as a fraction of available pore spaces.

Finally, ground and polished sections of the bone-endoprosthesis interface were then stained with Sanderson^™^ rapid bone stain and analyzed using a light microscope (Nikon Eclipse Ni, Tokyo, Japan).

### Statistical analysis

Statistical differences in bone ingrowth of this study group was compared to our previous study groups [15] using the Student’s t-test. A p-value of less than or equal to 0.05 was considered statistically significant.

## Results

**Fig 2:**
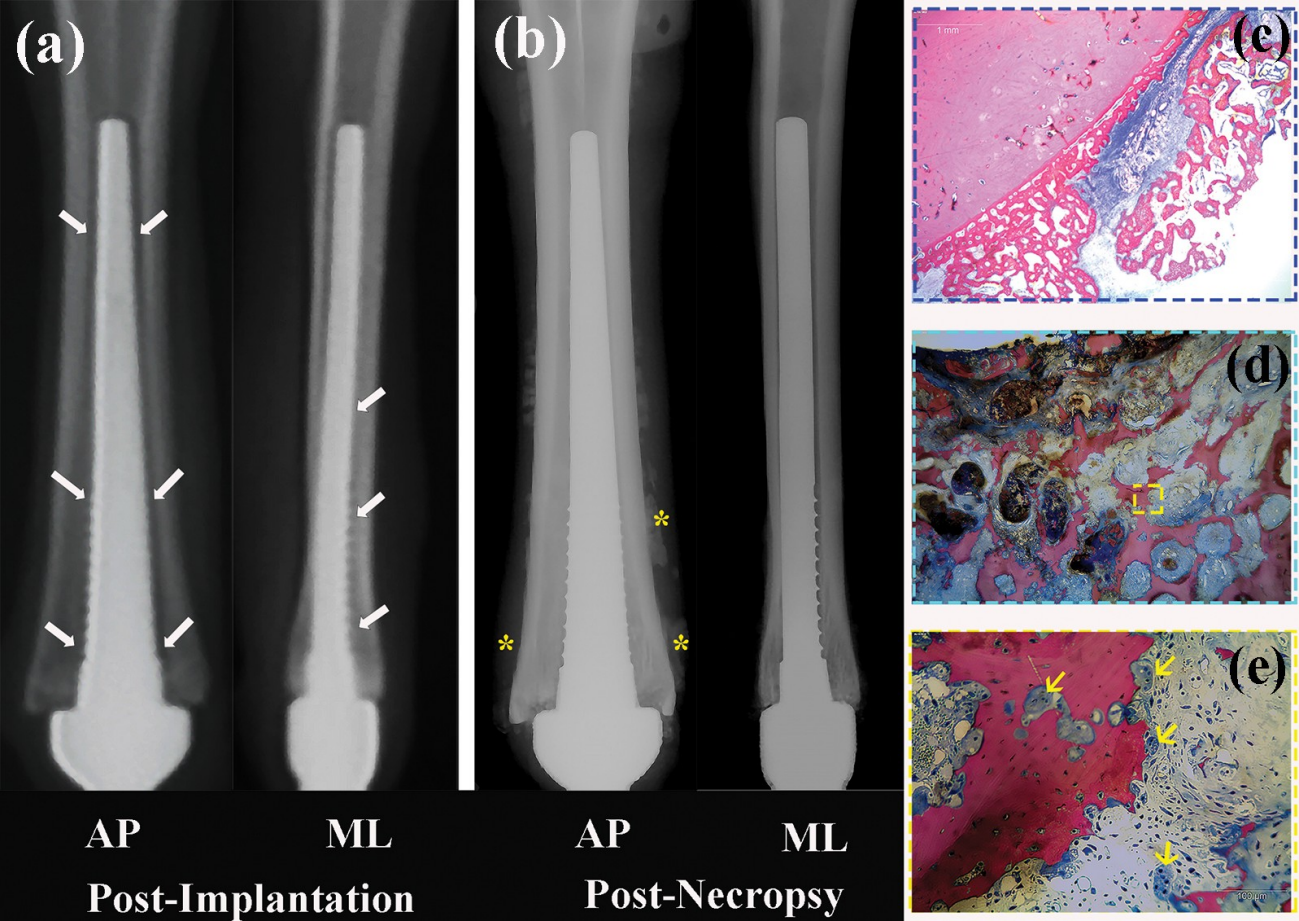
AP and ML radiographs of the metacarpal from the animal that developed clinical signs of infection ((a)—taken Post-Surgery and (b)–taken Post-Necropsy). Radiographs showed pronounced radiolucent zones medial, lateral and anterior to the endoprosthetic device (white arrows) and show periosteal reactive bone responses (*), a common sign of infection. Subsequent histological imaging verified a sub-periosteal infection (cross-sections stained with Sanderson Rapid Bone stain ((c), (d), and (e)); Pink-mineralized tissue and blue-fibrous tissue). Woven-bone exiting the periosteal surface is the reactive bone formation equivalent to sequestrum formation seen in the presence of chronic infection. Yellow arrows point to osteoclasts.

Of the eight-sheep implanted, only five animals survived to the 24-month endpoint, and were included in this study. Three animals did not survive to the 24-month end point and were thus excluded from further analyses. In the first animal, although the endoprosthetic stem seemed securely fixed when impacted into the medullary canal at time of surgery, anteroposterior radiographs taken at “Time 0” **(Fig 2(A))** confirmed that the endoprosthesis was undersized relative to the dimensions of the medullary canal. At the time of necropsy (28 days later), there was both radiological and histological evidence of periosteal changes **(Fig 2(B))** and sequestrum formation **(Fig 2(C)–2(E)),** indicative of infection. Because of the absence of initial fixation and stability, this animal was excluded from further analyses. As previously stated [19], a second sheep was euthanized early due to a failed tenodesis of the hoof flexors and extensors. At 15-months, a third animal became infected and was removed from the study group leaving 5 animals to complete the 24-month analysis of bone-device integration and adaptation.

**Fig 3:**
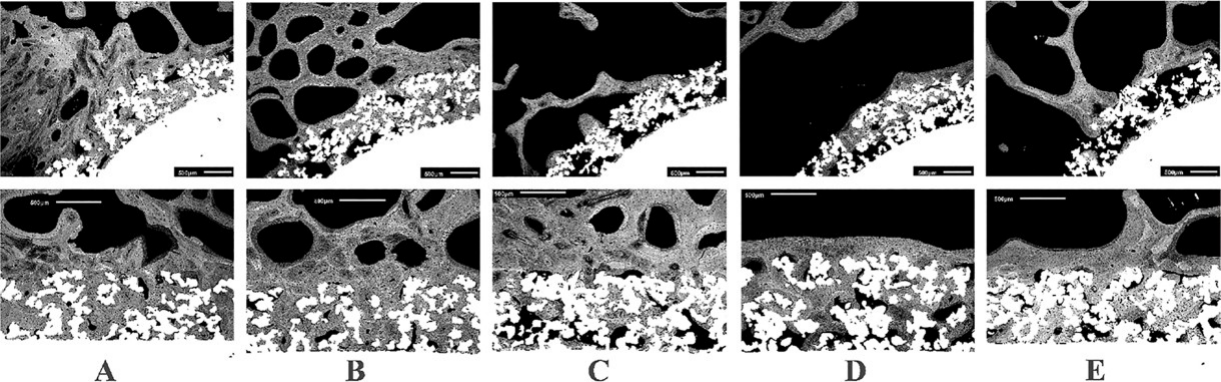
Representative BSE photomicrographs showing bone ingrowth into the porous-coated region of the five animals (X50 original magnification). In cross-sections of Animals A, B, and C, bone ingrowth is pronounced and mineralized. In cross-sections of Animals D and E, while there is bone ingrowth, there is also evidence of periprosthetic resorption, which is likely due to adaptation of stress shielded bone. Scale bar = 500μm. Porous coating; gray = bone; black = soft tissues, marrow, and cellular components.

The remaining 5 animals showed that the commercially pure titanium porous coating had significant bone ingrowth (**Fig 3**). Each of the 5 animals (Animal A-Animal E) is represented with a low power image (top) and a higher power image (bottom). Some device-coating interfaces showed bone ingrowth that was pronounced and mineralized **(Fig 3A, 3B and 3C)**, while others showed evidence of periprosthetic bone resorption that may be the result of stress shielding **(Fig 3D and 3E)**. On average, the percent of the pore volume filled with bone at 24-months post-implantation was 59.9 ± 24.5% (±STD), not statistically different from the 71 ± 8% previously reported at 12-months [15].

**Fig 4:**
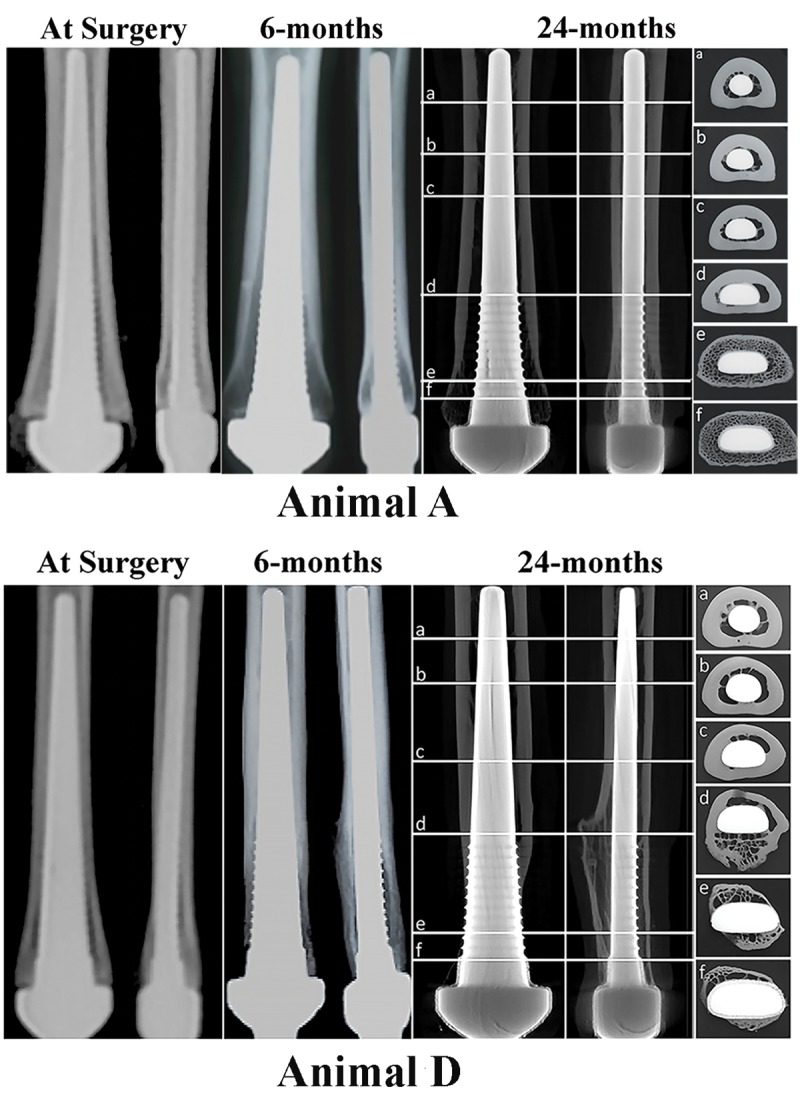
Serial radiographs taken at post-surgery (“Time 0”), at 6-month follow-up and at necropsy, 24-months post-implantation for Animal A and Animal D. Each panel shows AP view and ML view for each animal. Cross-sectional images in the far-right panel were acquired from μ-CT taken at 24-months post-surgery. Sections (a) from the proximal region through section (f) at the most distal region. Radiographs from Animal “A” show an endoprosthesis with an ideal end bearing fitting; Radiographs from Animal “D” show an endoprosthesis that was tightly bound in the proximal medullary canal. This resulted in distal bone resorption.

In Animal D (**Figs 3D and 4, Animal D**), the periprosthetic bone tissue was completely resorbed on the medial and lateral sides, but the distal region of the endoprosthesis appeared to be supported by bone ingrowth into the pores in the anterior and posterior regions. Regardless of this pronounced periprosthetic resorption, on average 51% of the pore spaces were filled with viable bone tissue in all animals (Table 1). Moreover, SEM data indicated that, at the anterior and posterior sites in most of the animals, the bone structure had remodeled into densely packed trabeculae with osteons, demonstrating “corticalization” of the distal cancellous bone at the epiphyseal/metaphyseal junction of the metacarpal.

**Table 1:** Ingrowth (%) data for the individual animal.

	Average ingrowth (%)	SD (%)
Animal A	77	21
Animal B	66	27
Animal C	47	18
Animal D	51	25
Animal E	58	22

**Fig 5:**
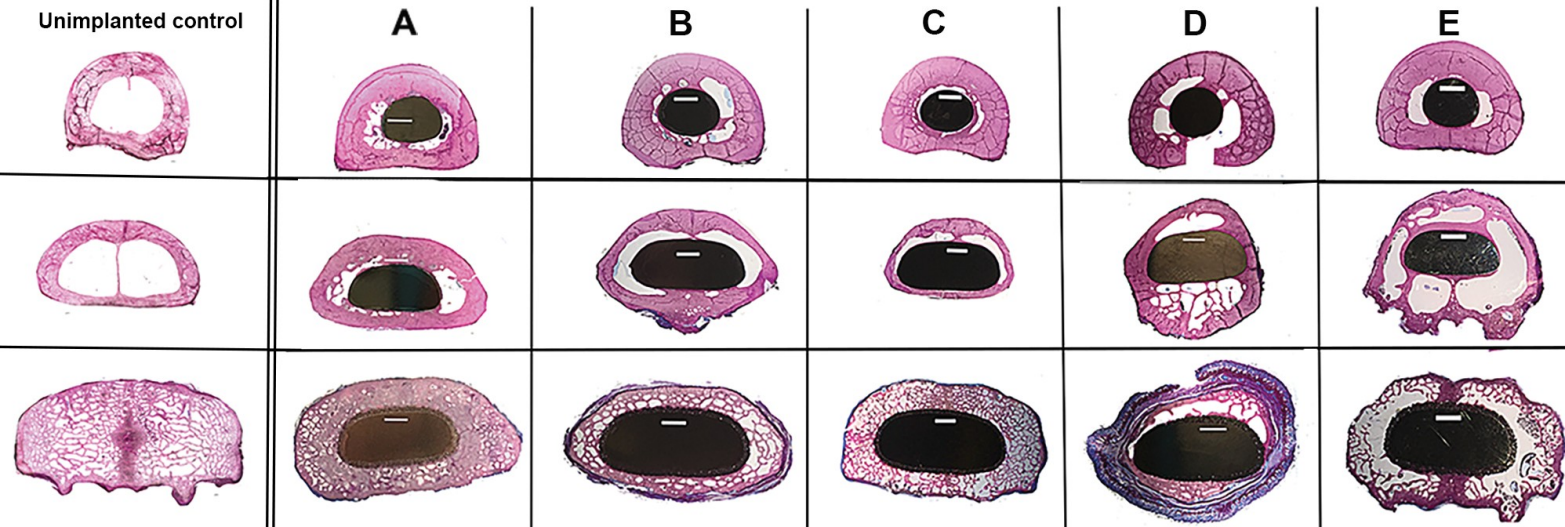
Cross-sectional images of a non-implanted control animal with representative bone-endoprosthesis cross-sections of the five experimental animals (A-E), stained with Sanderson Rapid Bone Stain. Sections were taken from different regions along the length of the endoprosthesis. The top row shows images from the smooth region of the endoprostheses, see **Fig 4,** Regions (a) or (b) or (c). The middle row shows images from the fluted regions of the endoprostheses, see **Fig 4**, Regions (d) or (e). The bottom row shows images from the porous coated region, of the endoprostheses, see **Fig 4**, Region (f). At surgery, the porous coated region was impacted into the cancellous metaphyseal bone. Pink = mineralized tissue (bone), black = endoprosthesis and blue = fibrous tissue. Scale bar = 3mm.

**Fig 6:**
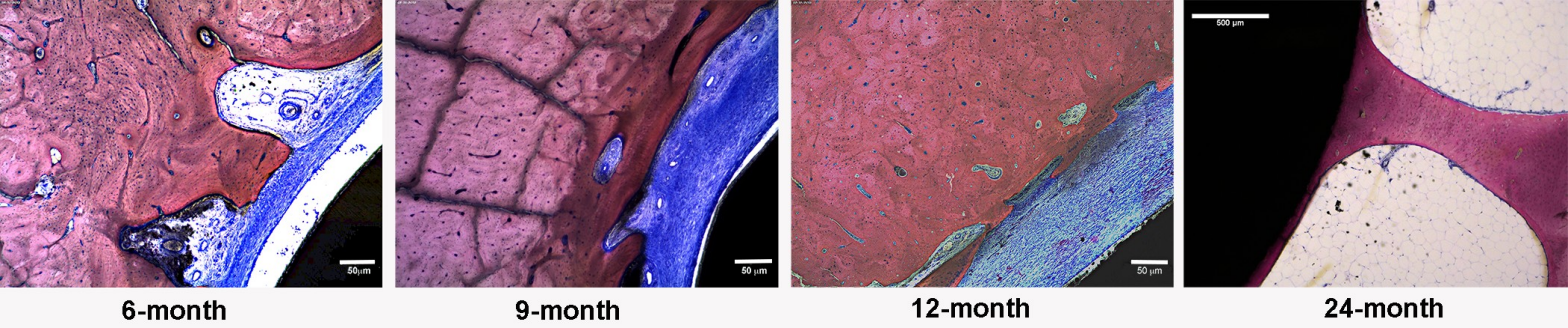
Photomicrographs showing the proximal end of the endoprosthesis-bone-interface. In 6-, 9-, and 12-month animals, the majority of the sites showed an interposing fibrous capsule (blue) between the implant (black) and the bone (pink).

**Fig 7:**
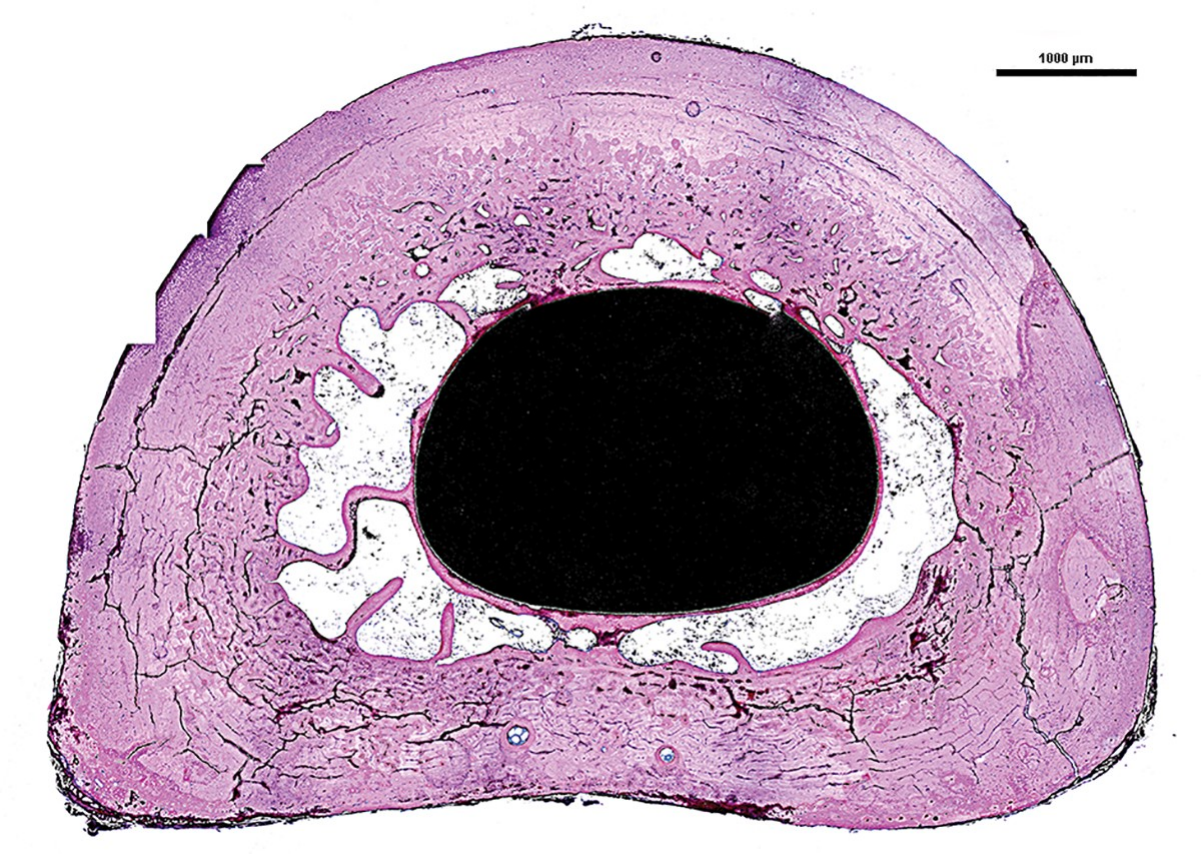
Photomicrograph of representative bone-endoprosthesis stem interface stained with Sanderson rapid bone stain: Black = endoprosthesis, pink = bone and blue = fibrous tissue. Scale bar = 1000 μm.

These ingrowth findings were further supported by the histology, μ-CT and radiographic data. Representative sets of radiographic images are given in **Fig 4, Animal A and Animal D**. These images clearly show the adaptive bone remodeling along the length of the endoprosthesis. When the proximal stem was not tightly bound within the medullary diaphysis (**Fig 4, Animal A**), the distal cancellous bone hypertrophied and distal bone preservation was achieved (**Fig 5A–5C**). However, when the endoprosthesis was tightly bound in the proximal diaphyseal canal (mid canal), over time, bone adaptation occurred and led to pronounced distal bone resorption/atrophy (**Fig 4, Animal D; Fig 5D–5E**). Moreover, axial images showed that, throughout the length of the endoprosthesis, there was no intervening fibrous encapsulation between the endoprosthesis and the periprosthetic bone (**Figs 5 and 6**). A closer look at the unbound endoprosthesis stem in the proximal medullary canal **(Fig 7)** revealed pseudo-cortex bone encapsulation (a thin shell of approximately 100 μm in thickness of unstructured bone) of the endoprosthesis’ grit blast surface. This pseudo bone appeared stabilized by tendril like bone structures connecting this pseudo-cortex to the endosteal bone surface.

## Discussion

The tested hypothesis, which stated that there would be no difference in bone-ingrowth between 12–24 months was statistically supported. Second part of the hypothesis, which stated that the bone adaptation (modelling and remodeling) would be completed by 12, was not supported. Radiographically data indicated that, when the endoprosthesis was bound at the proximal end, bone adaptation continued beyond 12 months.

The overall results from this study showed that the distally porous coated endoprosthetic design, used in this study, can ensure the distal fixation of the percutaneous OI device to residual bone and thereby prevent excessive distal bone resorption in the sheep. When compared to reported clinical bone absorption outcomes of human amputees fitted with osseointegrated devices [10, 23, 24], the current results clearly support the premise that distal loading of the bone with a short (10mm) porous structured titanium surface exposed to the endosteum of the medullary canal, can prevent distal bone atrophy and may produce stable long-term fixation.

For any permanent percutaneous OI device to be functionally successful in the human clinical application, the device design i.e. the shape, material, and location of fixation features, should allow for load sharing between the endoprosthesis and periprosthetic bone to accommodate bone preservation and to prevent stress shielding. Endoprosthetic designs that shield distal bone from strain, or sub-optimal surgical placement of OI devices, leads to adverse skeletal remodeling due to mechanotransduction phenomena and may endanger the their longevity [25]. In this study, the endoprosthesis was specifically designed to transfer load to the distal transected bone end to minimize distal bone adaptation and allow overall bone preservation. In three animals (A, B and C) the endoprosthesis functioned as designed and excellent maintenance of distal cancellous and cortical bone structures was observed over the 24-month study period. However, in two animals (D and E), high degrees of periprosthetic bone resorption were observed. In both animals, this outcome could be attributed to the less than optimal surgical placement of the endoprosthesis due to lack of endoprosthesis size availability and resulting in proximal endoprosthesis binding and distal stress shielding with bone resorption.

Bone is a biologically active tissue and is constantly undergoing a remodeling process to the extent that, in the skeletally mature person, most of the boney structures are completely replaced every ten-years. Within the bone replacement process the structure of bone adapts in response to the stresses and strains, or lack thereof, placed upon the skeletal architecture. This effect was first described by Julius Wolff in the 19th Century and is now known as Wolff’s Law [26, 27]. However, some have suggested that the better term for this effect is “bone functional adaptation in response to loading” [28]. Based on this theory, any portion of bone that is shielded from forces by an implanted device has a tendency to resorb over time [29, 30]. These effects have been observed particularly with the OPRA device [31, 32], and in Animals D and E of this study.

It could be argued that an inflammatory microenvironment within the periprosthetic soft-tissue may have promoted periprosthetic bone tissue resorption in Animals D and E, where Grade 3 infections were noted [19]. This appears unlikely in that Animal A, also had a Grade 3 infection, during which the cancellous bone structure was preserved and bone resorption was resisted. Moreover, the serial radiographs showed that bone resorption was radiographically visible at 6-months post-surgery in Animals E and D, at a time when downgrowth was graded as “zero” thus eliminating inflammation as a part of this resorptive process. It is strongly believed that the reason for the bone resorption in these animals is the loading position of the endoprosthesis in the bone and consequent stress shielding.

As stated, the distal endoprosthesis region was coated with porous structured titanium to ensure distal bone ingrowth and fixation. Although a wide variety of porous surfaces have been designed for biological fixation to titanium, it was felt that pore sizes ranging between 30–800 μm would be ideal for predictably secure bone ingrowth [33]. The titanium porous coating that was used in this study was a unique coating type and had a bi-modal pore-size distribution (major cross-sectional diameter of 32 ± 17 μm and 257 ± 87 μm) with approximately 60% porosity. As state, at 24-months, a high degree of ingrowth was observed, with approximately 60% of the pore space filled with bone tissue. Interestingly, at 24-months, healthy bone was found within the pores of animals that had experienced dramatic periprosthetic bone resorption. The question remains whether the pronounced resorption observed would eventually over extended time proceed to further loss of the bone previously deposited within the pores. This may subsequently destabilize the bone-endoprosthesis construct. It is not felt that this requires further study because proper endoprosthesis design and surgical positioning should obviate this problem. This is further supported by the observation that when the bone was firmly seated on the collar, the distal cancellous metaphyseal bone structure was maintained **(Fig 5A–5C).**

It is now known that any adaptive bone remodeling activity can be predicted by early radiographic imaging. This was seen in the prospective 6-monthly radiographs used to follow the adaptive periprosthetic bone remodeling around the endoprostheses **(Fig 4, Animal D).** The high degree of distal bone resorption and proximal bone hypertrophy that was observed in these animals continued to persist throughout the 24-month study period. The most ideal case was sheep A, where the endoprosthesis was fixed only distally and was seated at the collar, and the line-to-line fit of the proximal stem stabilized the endoprosthesis, allowing for osseointegration. This appeared to have allowed the host bone tissue to maintain its’ initial bone volume and cancellous configuration and avoided resorption. Again, this situation is not surprising based on Wolff’s Law of bone adaptation and the Utah paradigm of skeletal remodeling [28, 34–36].

The most important and clinically relevant radiographical finding was that the radiographic changes seen at 6 months continued to progress with increasing endoprosthesis *in situ* time **(Fig 4, Animal A and Animal D).** This observation indicated that adverse bone responses due to suboptimal endoprosthesis design and placement can be spotted early, and this could possibly lead to early intervention to avoid further resorption. Based on the radiographic signs of bone remodeling, it was concluded that distal bone attachment without any proximal endoprosthesis binding (as observed in the case A) could produce an ideal percutaneous OI system, which has the potential to maintain bone volume over extended time.

Histological observation of the proximal and therefore unloaded bone-endoprosthesis interface revealed, in axial section, that the endoprosthetic stem was encapsulated with a thin shell of unstructured bone tissue **(Fig 7).** Perhaps, this may have added stability to this endoprosthesis system and could have contributed to its success.

As seen in sheep radiographs **(Fig 4, Animal A),** distal loading and distal fit that avoids complete proximal fill of the medullary canal should be considered an essential element of design and is necessary for conservation and preservation of host bone. This observation is also supported by the previous reports of various clinically implemented endoprosthesis designs [37–39]. In one study, Werner and his co-authors concluded that a partially coated prosthesis with short segmental distal intramedullary fixation ensured more physiologically adaptive remodeling compared to the fully coated endoprosthesis systems. Furthermore, although clinical data on the Compress® (Zimmer Biomet, Warsaw, IN) mega prosthesis show that the design is susceptible to novel forms of bone failure [40–42], they also demonstrate pre-stress applied to the bone by the anchorage component at the distal osteotomy, encourages distal bone hypertrophy [38, 39].

Although complete press-fit of the endoprosthesis within the medullary canal is not considered necessary for bone preservation, the initial stability of the endoprosthesis within the intramedullary canal must be achieved in order to obtain secondary stability via osseointegration. The porous coating length (10 mm) used within this study was sufficient for secondary distal stabilization of the endoprosthesis by ingrowth. However, this length may need to be revised when applied to human devices and based on the morphology of the medullary canal and the anticipated loading environment.

Although the OI design tested in this study will help to establish design principles for percutaneous OI prosthesis, it is possible to improve the rate of osseointegration by other means. This might be achieved by stimulating the production of endogenous chemokines and cytokines or by local delivery of exogenous chemokines and cytokines that improve osseointegration. Osteoinductive materials such as hydroxyapatite coatings also might stimulate osseointegration. Instrumentation that will help to achieve an extract press fit and distal endoprosthesis seating, without causing any heat-induced bone necrosis, are important for eventual long-term device success rates.

The limitations of this study are four-fold. The first is the ability to legitimately transfer animal data to the human condition. It is well known that the healing, either bone-to-endoprosthesis or skin-to-endoprosthesis, is a complex process that involving various coordinated biological systems. There are differences in bone metabolic pathways and rates of these processes in animals when compared to the human. However, the literature indicates that the sheep bone model is a very acceptable model for bone-ingrowth studies [17]. Secondly, this study only used 5 animals; this small sample size reduced our ability to reach totally reliable conclusions. Thirdly, although not yet reported in the percutaneous OI prosthetic literature, osteolysis may also contribute to distal bone resorption at the porous coated regions; intuitively this seems unlikely. Wear particle debris could conceivably be generated at the trunnion joint linking the artificial limb to the endoprosthesis. However, skeletal docking systems have no moving parts to generate particulate debris and the foreign body reaction termed “particle disease” associated with resorption and clinical loosening of total joint replacement devices in arthroplasty patients. Finally, in our animals, the inflammatory microenvironment and the variable microbiome of the percutaneous stoma could have influenced periprosthetic bone adaptation. Since the tissues were embedded in PMMA, it was not possible to carry-out further histological analyses that might further clarify the inflammatory status of the periprosthetic tissue. Future studies should include molecular methods such as complete RNA sequencing or PCR array studies to parse out this possibility.

## Conclusion

End-loading ensured by bone ingrowth into the distal porous coating of the endoprosthesis has proven to be an effective design principle. The data have clearly demonstrated that the distal end-loading of the residual bone, coupled with adequate primary endoprosthesis stability, leads to conservation of the distal cortical bone with subsequent distal bone hypertrophy. Based on this result, and the evidence of adverse cortical remodeling in some other human OI endoprosthesis designs, “distal fit without proximal fill” should be used as a guide for future endoprosthesis designs. This principle should be confirmed in future studies.
